# Tenecteplase versus standard care for minor ischaemic stroke according to the baseline occlusion status in TEMPO-2 trial

**DOI:** 10.1093/esj/aakag111

**Published:** 2026-09-14

**Authors:** Silja Räty, Daniel Strbian, Nishita Singh, Shabnam Vatanpour, Liisa Tomppo, Aravind Ganesh, Bruce C V Campbell, Mark W Parsons, Michael D Hill, Shelagh B Coutts

**Affiliations:** Department of Neurology, Helsinki University Hospital and University of Helsinki, Helsinki, Finland; Department of Neurology, Helsinki University Hospital and University of Helsinki, Helsinki, Finland; Department of Internal Medicine, Section of Neurology, Rady Faculty of Health Sciences, University of Manitoba, Winnipeg, Manitoba, Canada; Departments of Clinical Neurosciences, Radiology and Community Health Sciences, Cumming School of Medicine, Hotchkiss Brain Institute, University of Calgary, Calgary, Canada; Department of Neurology, Helsinki University Hospital and University of Helsinki, Helsinki, Finland; Departments of Clinical Neurosciences, Radiology and Community Health Sciences, Cumming School of Medicine, Hotchkiss Brain Institute, University of Calgary, Calgary, Canada; Department of Medicine and Neurology, Melbourne Brain Centre at the Royal Melbourne Hospital, University of Melbourne, Parkville, Victoria, Australia; Department of Neurology, Liverpool Hospital, Liverpool, Australia; School of Clinical Medicine, South Western Sydney Campus, University of New South Wales, Liverpool, Australia; Sydney Brain Centre, Ingham Institute of Applied Medical Research, Liverpool, Australia; Departments of Clinical Neurosciences, Radiology and Community Health Sciences, Cumming School of Medicine, Hotchkiss Brain Institute, University of Calgary, Calgary, Canada; Departments of Clinical Neurosciences, Radiology and Community Health Sciences, Cumming School of Medicine, Hotchkiss Brain Institute, University of Calgary, Calgary, Canada

**Keywords:** arterial occlusion, cerebrovascular disorders, ischaemic stroke, minor stroke, tenecteplase, thrombolysis

## Abstract

**Introduction:**

Patients with minor ischaemic stroke (NIHSS ≤ 5) and intracranial occlusion or focal perfusion abnormality ≤ 12 h of onset did not benefit from intravenous tenecteplase compared to non-thrombolytic standard care (control) in the Tenecteplase vs Standard of Care for Minor Ischaemic Stroke with Proven Occlusion (TEMPO-2) trial. We tested whether tenecteplase was associated with better outcomes in subgroups defined by baseline vessel occlusion status.

**Patients and methods:**

In this secondary analysis of the randomised, controlled, blinded TEMPO-2 trial, patients were divided into 3 subgroups: complete occlusion (revised arterial occlusive lesion score 0), near occlusion (score 1) and no visible occlusion (focal perfusion abnormality present). The primary outcome was return to pre-stroke neurological functioning or better, assessed with 90-day mRS. Outcomes were analysed using a generalised linear model adjusted for age, sex, onset-to-randomisation time and baseline stroke severity.

**Results:**

Of 881 patients, complete occlusion was present in 218 in the control and 216 in the tenecteplase arm, near occlusion in 102 and 92 and no visible occlusion in 130 and 123, respectively. At baseline, there were longer treatment delays in the control arm with near occlusion, and older age, more hypertension and higher creatinine levels in the tenecteplase arm with complete occlusion. We found no difference in the primary outcome between tenecteplase and control in any subgroup (no visible occlusion: adjusted RR 1.03 [95% CI, 0.90–1.18]; near occlusion: adjusted RR 0.85 [95% CI, 0.71–1.01]; complete occlusion: adjusted RR 1.00 [95% CI, 0.89–1.12]).

**Discussion and conclusion:**

There was no difference in outcome between tenecteplase and standard care for patients with mild stroke based on their baseline vessel occlusion status.

**Trial registration:**

Clinicaltrials.gov: NCT02398656.

## Graphical abstract

**Graphical Abstract:**
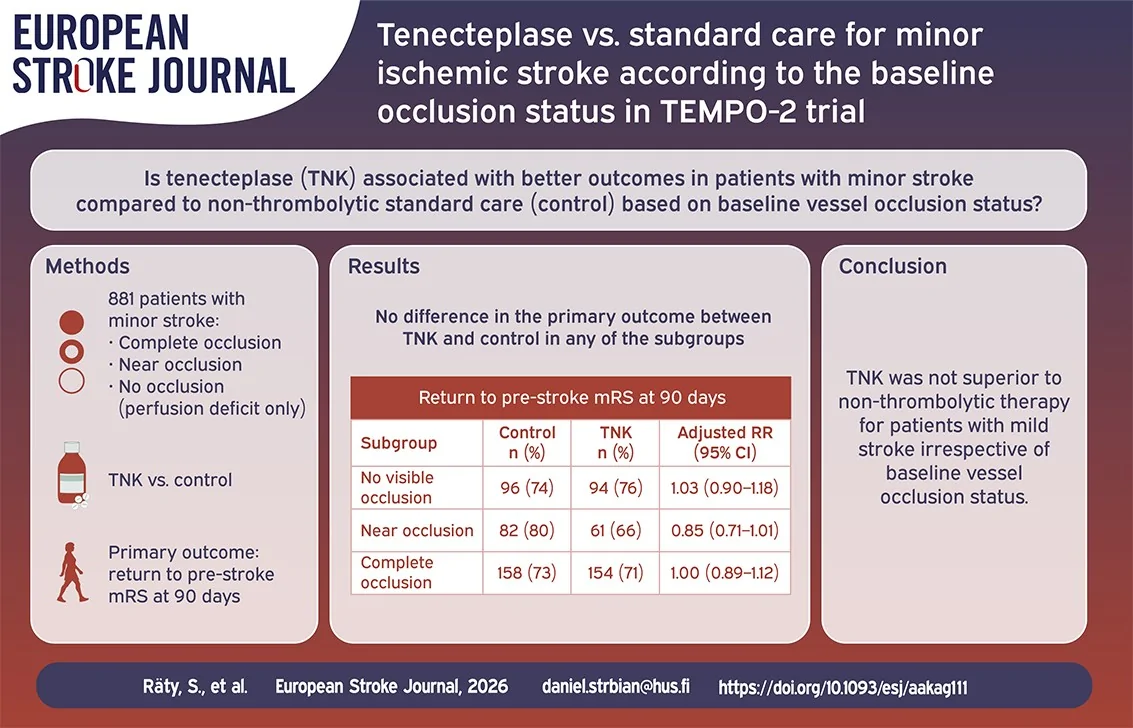

## Introduction

Acute ischaemic stroke (AIS) with mild symptoms presents a clinical challenge, as the optimal management strategy remains debated. While intravenous thrombolysis (IVT) is recommended for patients with disabling deficits independent of symptom severity,^1^^,^^2^ those with mild symptoms represented only 10% of individuals included in the landmark IVT trials, and patients with non-disabling symptoms were largely excluded.^3^ Since then, randomised controlled trials (RCTs) comparing IVT and non-thrombolytic therapy (usually single antiplatelet therapy [SAPT] or dual antiplatelet therapy [DAPT]) in patients with mild stroke symptoms have been either neutral^4–7^ or demonstrated non-inferiority of DAPT.^8^ On the other hand, DAPT as a secondary prevention strategy has been shown to reduce stroke recurrence without significant increase in bleeding complications when administered to patients with AIS and mild symptoms.^9–11^

Most RCTs on IVT in patients with mild stroke have not required imaging evidence of vessel occlusion for inclusion.^4–6^^,^^8^ In contrast, the neutral randomised controlled Tenecteplase vs Standard of Care for Minor Ischaemic Stroke with Proven Occlusion (TEMPO-2) trial comparing IVT with tenecteplase to non-thrombolytic therapy for patients with minor stroke enrolled only participants with either confirmed occlusion or arterial perfusion deficit.^7^ For patients with moderate-to-severe stroke with visible vessel occlusion, IVT has been superior to no reperfusion therapy in achieving successful recanalisation, a major predictor of outcome in AIS, particularly in more distal occlusions.^12^^,^^13^ Understanding how baseline vessel patency influences response to thrombolysis vs non-thrombolytic therapy in patients with mild deficits could help to identify patients potentially benefiting from IVT and guide more tailored treatment decisions.

In this secondary analysis of data from the TEMPO-2 trial, we aimed to determine whether tenecteplase was associated with better functional outcomes compared to non-thrombolytic standard care in patients with minor stroke stratified by baseline vessel occlusion status.

## Methods

This is a prespecified secondary analysis of the TEMPO-2 trial - a multicentre, prospective, randomised, open-label with blinded endpoint assessment (PROBE), controlled trial testing the superiority of intravenous tenecteplase (0.25 mg/kg) over non-thrombolytic treatment in patients with minor ischaemic stroke. The trial was conducted at 48 hospitals in Australia, Europe, North America, South America and Asia. The protocol, statistical analysis plan and the main results of the trial have been previously published.^7^^,^^14^ The trial was approved by local ethics boards, and written informed consent was obtained from all patients or their representatives.

The clinical selection criteria included age 18 years or older, mild symptom severity defined as NIHSS score of 0–5 at baseline, pre-stroke mRS score of 0–2, presentation within 12 h of last seen well and no contraindication (except for time window) for IVT. The imaging criteria entailed either direct or indirect evidence of intracranial occlusion, ie, angiography-verified occlusion or deficit in perfusion imaging compatible with presenting symptoms. Perfusion deficit was defined as any area of focal perfusion abnormality in CT or MR perfusion visually identified by a local investigator. No region of already established infarction concordant with the acute symptoms and an Alberta Stroke Program Early CT score^15^ 7 or higher were required.

Patients were randomised (1:1) to receive intravenous tenecteplase 0.25 mg/kg or standard non-thrombolytic treatment (control). The choice of antithrombotic therapy in the control arm was left to the discretion of the treating physician following guideline recommendations and consisted of either SAPT, DAPT or anticoagulation. Other treatment was similar in both arms, including standard stroke unit care. Either brain CT or MRI was performed at 24 h, and a subset of patients underwent CTA at 4–8 h, where recanalisation was defined as a revised arterial occlusive lesion (rAOL) score 2b–3.^12^

In this sub-study, we compared the impact of tenecteplase vs control on outcome stratified by vessel occlusion status. Based on the baseline imaging, we divided the patients into 3 groups: (1) complete occlusion (rAOL 0), (2) near occlusion (rAOL 1) and (3) no visible occlusion but focal perfusion abnormality. Imaging data were assessed centrally at the University of Calgary core laboratory by a neuroradiologist blinded to clinical information.

### Outcomes

The primary outcome of the TEMPO-2 trial and the current study was return to baseline neurological functioning or better (responder) as measured by mRS at 90 days, assessed by an investigator blinded to the treatment allocation. A sliding dichotomy approach was used to define a responder: for patients with pre-stroke mRS 0–1, it was return to mRS 0–1 at 90 days and for patients with pre-stroke mRS 2, it was mRS 0–2. Secondary outcomes included 90-day mRS 0–1 (excellent outcome), 90-day mRS 0–2 (functional independence), comparison of the mean 90-day mRS, NIHSS at day 5 (or at discharge if earlier), percent function on Lawton Instrumental Activities of Daily Living (IADL) Scale at 90 days,^16^ and quality of life according to the European Quality of Life score, 5 dimensions, 5 levels (EQ-5D-5L) at 90 days.^17^

Safety outcomes included sICH, defined as any type of new ICH according to the Heidelberg bleeding classification associated with neurological deterioration of NIHSS ≥ 2 points, in which the haemorrhage was judged to be the most important cause.^18^ In addition, stroke progression, recurrent stroke, all-cause mortality and proportion of patients receiving rescue endovascular thrombectomy for the index stroke were assessed.

### Statistical analyses

All outcomes were analysed in the intention-to-treat population stratified by the baseline vessel occlusion status. We used a generalised linear model with Poisson distribution, log link function and robust Huber–Sandwich standard error estimation to calculate risk ratios (RRs) and 95% CI using the control groups as the reference category. Mortality was assessed with the Cox model. The proportional hazards assumption was assessed graphically and statistically. All analyses were adjusted for a priori chosen set of variables of prognostic importance and because they were used in the randomised minimisation algorithm, including age, sex, baseline NIHSS and time from symptom onset to randomisation.

Heterogeneity of treatment effect was analysed using multiplicative interaction terms across the prespecified subgroups of age (≤80 vs > 80 years), sex (male vs female), time from symptom onset to treatment (≤4.5 vs > 4.5 h), occlusion location (LVO [internal carotid artery or the M1 segment of middle cerebral artery] vs MeVO [M2 or more distal segment of middle cerebral artery, anterior cerebral artery] vs vertebrobasilar [includes posterior cerebral artery]) if present, baseline NIHSS score, and whether symptoms had resolved before randomisation.

Missing data on the primary outcome for patients lost to follow-up were imputed as non-responders. No imputations were made for patients who withdrew consent (*n* = 2). For patients who were dead at 90 days, the worst possible score was imputed for EQ-5D-5L and Lawton IADL Index. We imputed missing values on the NIHSS score at 5 days or discharge using the last score carried forward principle. Other missing data were not imputed. No correction for multiplicity was performed. Analyses were conducted with STATA (version 18).

## Results

Altogether 886 patients were enrolled in TEMPO-2 and 5 were excluded from this analysis due to withdrawal of consent (*n* = 2) or lack of available or evaluable imaging (*n* = 3). Of 881 participants in the final cohort, 434 (49.3%) had a complete occlusion (tenecteplase 216; control 218), 194 (22.0%) a near occlusion (tenecteplase 92; control 102) and 253 (28.7%) no visible occlusion but focal perfusion abnormality (tenecteplase 123; control 130). In the complete occlusion subgroup, 16.8% had LVO, 76.0% had MeVO and 7.1% had vertebrobasilar occlusion, whereas the numbers were 15.5%, 77.3% and 7.2% in the near occlusion group, respectively. There were few baseline differences between the subgroups in age, previous hypertension, creatinine level and onset-to-treatment times (Table 1). Recanalisation was achieved by 130/364 (36%) of the patients with complete occlusion and by 48/153 (31%) with near occlusion (*P* = .36).

**Table 1:** Baseline characteristics stratified by vessel occlusion status.

	No visible occlusion (focal perfusion lesion only)			Near occlusion			Complete occlusion		
	Control	TNK	*P*	Control	TNK	*P*	Control	TNK	*P*
***n* (%)**	130 (51.4)	123 (48.6)		102 (52.6)	92 (47.4)		218 (50.2)	216 (49.8)	
**Demographics**									
**Age (y)**	74 (64–81)	74 (61–80)	.471	72 (62–82)	71 (61–78)	.588	70 (60–78)	72 (62–82)	**.021**
**Sex at birth**									
**Female**	46 (35.4)	46 (37.4)	.794	37 (36.3)	35 (38.0)	.882	97 (44.5)	106 (49.1)	.387
**Male**	84 (64.6)	77 (62.6)		65 (63.7)	57 (62.0)		121 (55.5)	110 (50.9)	
**Race**									
**Caucasian**	115 (88.5)	113 (91.9)	.682	80 (78.4)	75 (81.5)	.867	185 (84.9)	182 (84.3)	.512
**First nations**	0 (0.0)	1 (0.8)		1 (1.0)	2 (2.2)		4 (1.8)	1 (0.5)	
**Pacific Islander**	0 (0.0)	0 (0.0)		0 (0.0)	0 (0.0)		0 (0.0)	1 (0.5)	
**Asian**	10 (7.7)	6 (4.9)		14 (13.7)	10 (10.9)		18 (8.3)	24 (11.1)	
**Black**	1 (0.8)	0 (0.0)		3 (2.9)	3 (3.3)		3 (1.4)	3 (1.4)	
**Other**	4 (3.1)	3 (2.4)		4 (3.9)	2 (2.2)		8 (3.7)	5 (2.3)	
**Ethnicity**									
**Non-Hispanic**	129 (99.2)	123 (100.0)	1.000	97 (95.1)	90 (97.8)	.449	215 (98.6)	213 (98.6)	1.000
**Hispanic**	1 (0.8)	0 (0.0)		5 (4.9)	2 (2.2)		3 (1.4)	3 (1.4)	
**Medical history**									
**Hypertension**	80 (61.5)	72 (58.5)	.700	64 (62.7)	54 (58.7)	.659	117 (53.7)	138 (63.9)	**.032**
**Past smoking**	49 (37.7)	38 (30.9)	.290	39 (38.2)	40 (43.5)	.469	87 (39.9)	94 (43.5)	.496
**Hyperlipidaemia**	60 (46.2)	49 (39.8)	.374	34 (33.3)	39 (42.4)	.235	78 (35.8)	92 (42.6)	.169
**Diabetes mellitus**	33 (25.4)	24 (19.5)	.294	21 (20.6)	19 (20.7)	1.000	32 (14.7)	39 (18.1)	.366
**Previous stroke**	25 (19.2)	21 (17.1)	.745	19 (18.6)	13 (14.1)	.443	41 (18.8)	38 (17.6)	.804
**Atrial fibrillation**	18 (13.8)	21 (17.1)	.492	14 (13.7)	14 (15.2)	.839	46 (21.1)	56 (25.9)	.258
**Ischaemic heart disease**	27 (20.8)	17 (13.8)	.184	13 (12.7)	14 (15.2)	.681	33 (15.1)	38 (17.6)	.518
**Congestive heart failure**	4 (3.1)	4 (3.3)	1.000	3 (2.9)	0 (0.0)	.248	11 (5.0)	12 (5.6)	.834
**Chronic renal failure**	5 (3.8)	2 (1.6)	.448	4 (3.9)	7 (7.6)	.356	8 (3.7)	13 (6.0)	.273
**Peripheral vascular disease**	3 (2.3)	4 (3.3)	.716	4 (3.9)	3 (3.3)	1.000	8 (3.7)	6 (2.8)	.787
**Past ICH**	0 (0.0)	1 (0.8)	.486	0 (0.0)	0 (0.0)	.	1 (0.5)	2 (0.9)	.622
**Clinical presentation**									
**NIHSS score at baseline**	2 (1–3)	2 (1–3)	.745	2 (1–3)	2 (1–3)	.189	2 (1–3)	2 (1–3)	.414
**Symptoms resolved at time of treatment**	19 (14.6)	18 (14.6)	1.000	19 (18.6)	21 (22.8)	.483	29 (13.3)	42 (19.4)	.092
**Haemoglobin, g/L**	140 (129–151)	140 (130–153)	.953	140 (131–151)	142 (126–150)	.442	142 (132–152)	139 (129–149)	.095
**Glucose, mM**	6 (5–8)	6 (6–7)	.274	6 (6–7)	6 (5–8)	.717	6 (6–7)	6 (6–7)	.728
**Creatinine, uM**	83 (70–97)	81 (70–94)	.476	87 (75–101)	82 (67–97)	.741	83 (68–97)	84 (70–101)	**.040**
**ASPECTS baseline**	10 (10–10)	10 (10–10)	.382	10 (10–10)	10 (9–10)	.216	10 (9–10)	10 (9–10)	.922
**Onset to randomisation time, min**	227 (157–447)	261 (145–455)	.886	298 (186–456)	294 (151–397)	.422	276 (161–441)	299 (173–456)	.689
**Onset to ED time, min**	146 (71–337)	144 (84–375)	.743	190 (81–354)	166 (76–296)	.879	138 (68–337)	142 (72–335)	.775
**Onset to treatment time, min**	320 (174–510)	270 (153–463)	.060	336 (214–529)	302 (158–393)	**.029**	301 (173–485)	304 (180–465)	.324
**Occlusion location at baseline**									
**Large vessel occlusion** ^**a**^	0 (0.0)	0 (0.0)	.490	17 (16.7)	13 (14.1)	.594	33 (15.1)	40 (18.5)	.664
**Medium vessel occlusion** ^**b**^	0 (0.0)	0 (0.0)		76 (74.5)	74 (80.4)		169 (77.5)	161 (74.5)	
**Vertebrobasilar circulation** ^**c**^	0 (0.0)	0 (0.0)		9 (8.8)	5 (5.4)		16 (7.3)	15 (6.9)	
**Focal perfusion lesion only** ^**d**^	127 (97.7)	118 (95.9)		0 (0.0)	0 (0.0)		0 (0.0)	0 (0.0)	
**No occlusion or perfusion lesion detected**	3 (2.3)	5 (4.1)		0 (0.0)	0 (0.0)		0 (0.0)	0 (0.0)	

Abbreviations: ASPECTS = Alberta Stroke Program Early CT score; ED = emergency department; TNK = tenecteplase.

Data are *n* (%) or median (IQR). Race and ethnicity were self-reported; ethnicity was binary with Hispanic or non-Hispanic being the options. Significant differences between the treatment arms are bolded.

^a^Intracranial internal carotid artery, M1 segment of the middle cerebral artery.

^b^M2 or more distal segment of the middle cerebral artery or A2 or more distal segment of the anterior cerebral artery.

^c^Intracranial vertebral artery, basilar artery or branches or posterior cerebral artery (basilar artery occlusion *n* = 0).

^d^Only focal perfusion lesion in perfusion imaging but no visible occlusion in angiography.

There was no difference in the primary outcome between patients treated with tenecteplase and control in any of the subgroups (Table 2). Among patients with no visible occlusion, the primary outcome was achieved by 76.4% in the tenecteplase group and 73.8% in the control group (adjusted RR 1.03 [95% CI, 0.90–1.18]), whereas the respective numbers were 66.3% and 80.4% in the near occlusion subgroup (adjusted RR 0.85 [95% CI, 0.71–1.01]) and 71.3% and 72.5% in the complete occlusion subgroup (adjusted RR 1.00 [95% CI, 0.89–1.12]).

**Table 2:** Outcome comparison between tenecteplase and control stratified by vessel occlusion status.

	No visible occlusion (focal perfusion lesion only)				Near occlusion				Complete occlusion			
Outcome	Control (*n* = 130)	TNK (*n* = 123)	Unadjusted RR/RD/HR (95% CI)	Adjusted RR/RD/HR (95% CI)	Control (*n* = 102)	TNK (*n* = 92)	Unadjusted RR/RD/HR (95% CI)	Adjusted RR/RD/HR (95% CI)	Control (*n* = 218)	TNK (*n* = 216)	Unadjusted RR/RD/HR (95% CI)	Adjusted RR/RD/HR (95% CI)
**Responder at 90 d, *n* (%)**	96 (73.8)	94 (76.4)	RR: 1.03 (0.90, 1.19)	1.03 (0.90, 1.18)	82 (80.4)	61 (66.3)	**0.82 (0.69, 0.98)**	0.85 (0.71, 1.01)	158 (72.5)	154 (71.3)	0.98 (0.87, 1.11)	1.00 (0.89, 1.12)
**mRS 0–1 at 90 d, *n* (%)**	89 (69.0)	93 (75.6)	RR: 1.10 (0.94, 1.28)	1.08 (0.94, 1.25)	77 (75.5)	59 (64.1)	0.85 (0.70, 1.03)	0.87 (0.73, 1.05)	153 (70.5)	146 (67.6)	0.96 (0.84, 1.09)	0.98 (0.87, 1.11)
**mRS 0–2 at 90 d, *n* (%)**	108 (83.7)	106 (86.2)	RR: 1.03 (0.93, 1.14)	1.02 (0.93, 1.13)	89 (87.3)	70 (76.1)	0.87 (0.76, 1.00)	0.89 (0.78, 1.01)	192 (88.5)	175 (81.0)	**0.92 (0.84, 0.99)**	**0.93 (0.86, 1.00)**
**NIHSS score 0 at 5 d or discharge, *n* (%)**	68 (52.3)	76 (62.8)	RR: 1.20 (0.97, 1.49)^a^	1.19 (0.98, 1.45)	52 (51.0)	49 (54.4)	1.07 (0.82, 1.40)^b^	1.12 (0.86, 1.47)	106 (48.6)	122 (56.7)	1.17 (0.97, 1.40)^c^	1.13 (0.96, 1.34)
**Mean mRS score at 90 d**	1.25	1.11	RD: −0.13 (−0.48, 0.21)	−0.11 (−0.44, 0.21)	1.00	1.38	0.38 (−0.02, 0.78)	0.34 (−0.06, 0.73)	1.08	1.30	0.22 (−0.04, 0.49)	0.18 (−0.08, 0.45)
**Mean Lawton IADL % functioning**	87.4	91.2	RD: 3.76 (−2.75, 10.26)^d^	3.11 (−3.07, 9.29)	92.7	84.1	−**8.60 (**−**15.92,** −**1.28)**^**e**^	−**8.77 (**−**15.94,** −**1.60)**	92.0	84.7	−**7.29 (**−**12.09,** −**2.48)**^**f**^	−**6.40 (**−**11.07,** −**1.72)**
**Mean EQ-5D-5L index**	0.83	0.84	RD: 0.01 (−0.05, 0.07)^g^	0.004 (−0.05, 0.06)	0.87	0.79	−**0.08 (**−**0.15,** −**0.01)**^**h**^	−**0.08 (**−**0.15,** −**0.01)**	0.84	0.80	−0.03 (−0.08, 0.01)^i^	−0.03 (−0.08, 0.02)
**Mean EQ-5D-5L VAS**	75.5	75.5	RD: −0.06 (−5.49, 5.38)^j^	−0.16 (−5.57, 5.24)	79.4	68.9	−**10.50** **(**−**16.65,** −**4.35)**^**k**^	−**10.19 (**−**16.41,** −**3.97)**	74.8	72.7	−2.04 (−6.20, 2.13)^l^	−2.04 (−6.24, 2.15)
**Death at 90 d, *n* (%)**	2 (1.6)	5 (4.1)	HR: 1.60 (0.27, 9.56)^a^	1.63 (0.27, 9.94)	0 (0.0)	6 (6.5)	NA	NA	3 (1.4)	9 (4.2)	3.13 (0.85, 11.57)	3.42 (0.91, 12.87)

Abbreviations: EQ-5D-5L = European Quality of Life score, 5 dimensions, 5 levels; HR = hazard ratio; Lawton IADL = Lawton–Brody Instrumental Activities of Daily Living Scale score; RD = risk difference; RR = risk ratio; TNK = tenecteplase; VAS = visual analogue scale.

Analyses were adjusted for age, sex at birth, baseline NIHSS score and onset to randomisation time; as the proportional odds assumption was not met, an ordinal shift analysis is not presented. Significant differences between the treatment arms are bolded.

^a^ *n* = 251. ^b^ *n* = 192. ^c^ *n* = 433. ^d^ *n* = 239. ^e^ *n* = 186. ^f^ *n* = 422. ^g^ *n* = 237. ^h^ *n* = 185. ^i^ *n* = 420. ^j^ *n* = 235. ^k^ *n* = 183. ^l^ *n* = 418.

**Figure 1:**
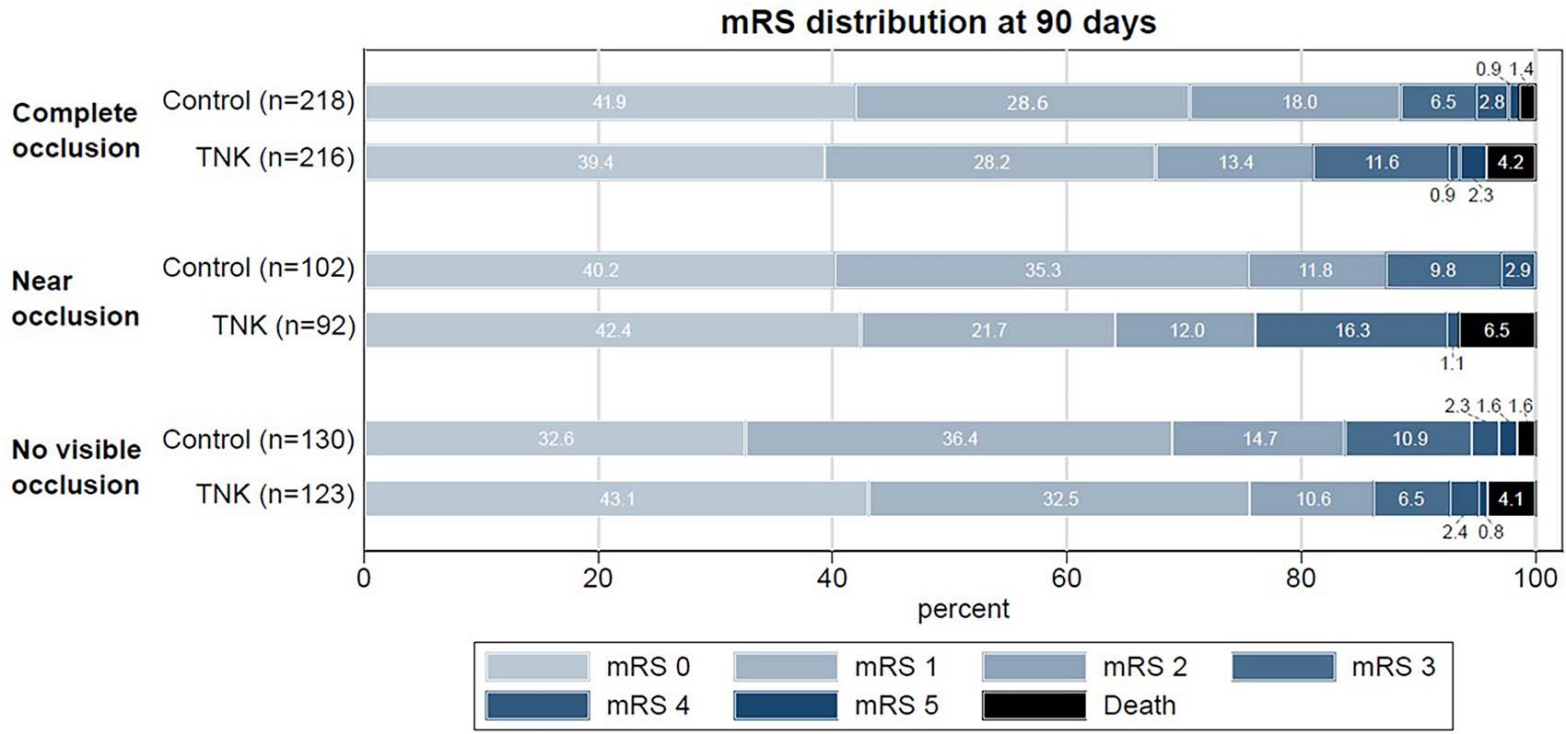
Distribution of the 90-day mRS in patients treated with tenecteplase vs control stratified by vessel occlusion status. Abbreviation: TNK = tenecteplase.

Regarding the secondary efficacy outcomes, 90-day mRS 0–2 occurred less often in the tenecteplase arm compared to control among the patients with complete occlusion (81.0% vs 88.5%, adjusted RR 0.93 [95% CI, 0.86–1.00]; Table 2, Figure 1). The control arm achieved better quality of life metrics and a higher Lawton IADL Scale score in the near occlusion group and the latter in the complete occlusion group. No other differences between the treatment arms were observed in any of the subgroups.

There were more cases of sICH in the tenecteplase arms stratified by the occlusion status, although the differences were not significant (Table 3). Mortality at 90 days (6.5% vs 0%, *P* = .010) and the frequency of serious adverse events (26.1% vs 13.7%, *P* = .045) were higher in the tenecteplase arm among patients with near occlusion. Other safety outcomes did not differ between the treatment arms (Table 3).

**Table 3:** Safety outcome comparison between tenecteplase and control stratified by vessel occlusion status.

	No visible occlusion (focal perfusion lesion only)			Near occlusion			Complete occlusion		
Safety outcomes	Control (*n* = 130)	TNK (*n* = 123)	*P*	Control (*n* = 102)	TNK (*n* = 92)	*P*	Control (*n* = 218)	TNK (*n* = 216)	*P*
**Serious adverse event, *n* (%)**	16 (12.3)	24 (19.5)	.124	14 (13.7)	24 (26.1)	**.045**	50 (22.9)	52 (24.1)	.821
**Stroke progression, *n* (%)**	5 (3.8)	6 (4.9)	.764	3 (2.9)	9 (9.8)	.071	25 (11.5)	20 (9.3)	.529
**Stroke recurrence, *n* (%)**	3 (2.3)	6 (4.9)	.323	5 (4.9)	3 (3.3)	.724	7 (3.2)	7 (3.2)	1.000
**sICH at 24 h, *n* (%)**	1 (0.8)	2 (1.6)	.613	0 (0.0)	2 (2.2)	.224	1 (0.5)	4 (1.9)	.214
**Rescue EVT, *n* (%)**	2 (1.5)	0 (0.0)	.498	2 (2.0)	5 (5.4)	.259	6 (2.8)	10 (4.6)	.322
**Death at 5 d, *n* (%)**	0 (0.0)	2 (1.6)	.235	0 (0.0)	2 (2.2)	.224	1 (0.5)	4 (1.9)	.214
**Death at 90 d, *n* (%)**	2 (1.6)	5 (4.1)	.272	0 (0.0)	6 (6.5)	**.010**	3 (1.4)	9 (4.2)	.088

Significant differences between the treatment arms are bolded. Abbreviation: TNK = tenecteplase.

**Figure 2:**
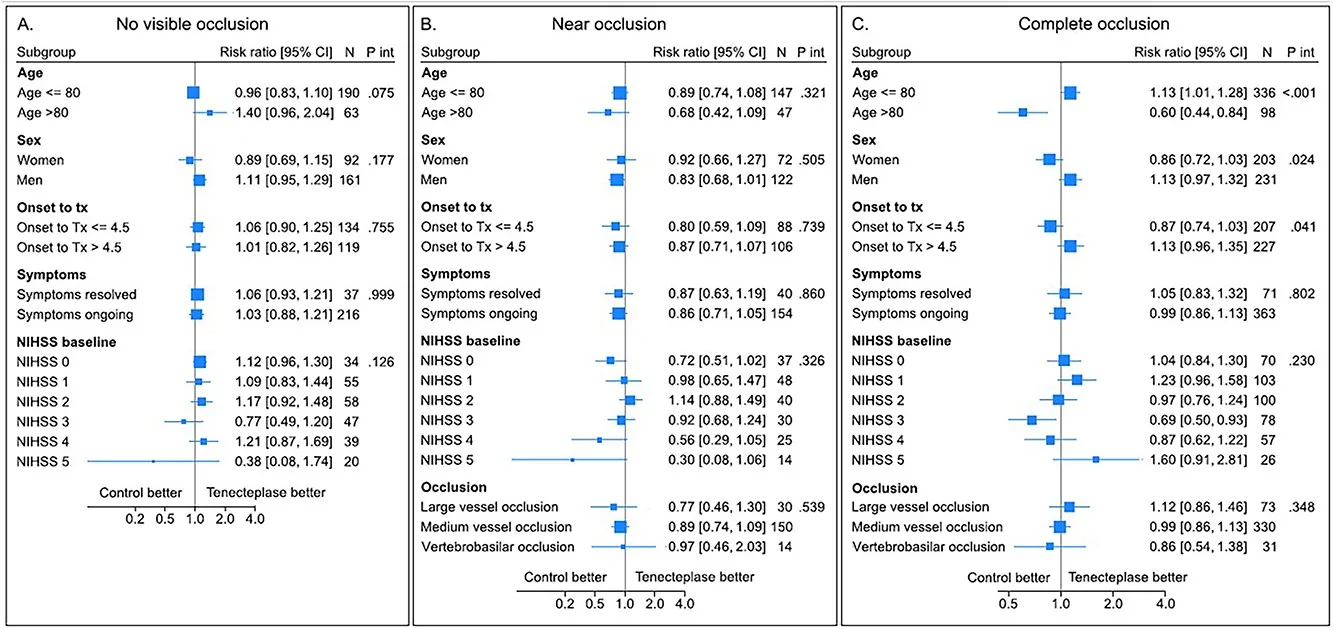
Forest plots of return to baseline neurological functioning or better (primary outcome) by subgroup, separately for each vessel occlusion status at baseline: (A) no visible occlusion (only focal perfusion abnormality), (B) near occlusion and (C) complete occlusion. RRs adjusted for sex at birth, baseline NIHSS score and onset to randomisation time. Abbreviations: RR = risk ratio; Tx = treatment.

There were no interactions in the no occlusion or near occlusion groups. Among the patients with complete occlusion, we found interactions of the treatment arm with age (*P* < .001), sex (*P* = .024) and time from onset (*P* = .041) (Figure 2). In further analyses, patients ≤ 80 years with complete occlusion were more likely to achieve the primary outcome after tenecteplase (adjusted RR 1.13 [95% CI, 1.01–1.28]), whereas the result was opposite for patients > 80 years (adjusted RR 0.60 [95% CI, 0.44–0.84]). In subgroup analyses of male and female patients and patients treated within or after 4.5 h of onset with complete occlusion, there were no significant differences between the treatments.

## Discussion

In this secondary analysis from the TEMPO-2 trial, there was no difference in return to pre-stroke functional outcome or better between tenecteplase and non-thrombolytic therapy based on the baseline vessel occlusion status among patients with minor AIS. In fact, a few secondary outcomes favoured non-thrombolytic therapy among patients with complete or near occlusion, whereas there were no differences between the treatments in the subgroup with no visible occlusion.

Previous RCTs on minor stroke have studied either alteplase^4^^,^^8^ or urokinase^5^^,^^6^ compared to non-thrombolytic treatment but have not required imaging evidence of vessel occlusion or perfusion deficit for trial inclusion. In the Antiplatelet vs R-tPA for Acute Mild Ischaemic Stroke (ARAMIS) trial, less than 10% of the participants had complete occlusion of the culprit vessel and 5% had confirmed LVO.^8^ Consistent with our study, neither baseline LVO, nor presence of > 50% culprit stenosis modified the neutral result between IVT and DAPT. In the Prourokinase for Mild Ischaemic Cerebrovascular Events (PUMICE) trial, only 1.3% of 1446 patients underwent vascular imaging before randomisation.^5^ The ThRombolysis of Urokinase for minor STroke (TRUST) and Potential of r-tPA for Ischaemic Strokes With Mild Symptoms (PRISMS) trials did not report data on the occlusion status, nor did an individual-patient data meta-analysis studying the effect of stroke severity on outcome in RCTs on intravenous alteplase vs control for patients with AIS.^3^^,^^4^^,^^6^

Our findings are consistent with the main results of the TEMPO-2 trial that did not demonstrate superiority of tenecteplase compared to non-thrombolytic therapy for patients with minor stroke, as well as with another secondary analysis of the trial that reported similar results irrespective of whether patients had disabling or non-disabling symptoms.^7^^,^^19^ In the present study, some secondary endpoints favoured the standard treatment in the complete and near occlusion subgroups. The tenecteplase-treated patients with near occlusion had higher 90-day mortality, more serious adverse events, poorer quality of life and a trend for poorer functional outcome compared to control, whereas the tenecteplase arm in the complete occlusion subgroup achieved less often mRS 0–2 and had poorer quality of life. As the number of patients in the subgroups was relatively small, the results should be interpreted with caution. However, the uniform findings in several outcome measures, including quality of life, suggest that they are not explained by only using metrics (mRS, mortality) that might lack sensitivity to detect differences among patients with mild neurological deficits. The excess mortality after tenecteplase in the near occlusion group cannot be solely explained by causes related to intervention, as most deaths (*n* = 4) occurred after the first 5 days and there were only 2 sICHs in the group.

In TEMPO-2, tenecteplase improved recanalisation compared to control among all patients with baseline occlusion who underwent control angiography (48% vs 22%), and among patients with LVO (48% vs 13%).^7^ As recanalisation has been identified as a major contributor to improved outcome,^13^ it was unexpected that outcome of patients receiving tenecteplase did not differ from control, not even in those with complete occlusion. However, the relationship between recanalisation and outcome has been mostly drawn from studies on LVO, infrequently present in patients with mild symptoms. In the post-hoc analysis of the ARAMIS trial, there was no difference in early neurological deterioration or functional outcome between alteplase and DAPT among patients with LVO, but these represented less than 10% of the study population.^20^ In the current study, LVOs constituted 17% of complete occlusions and 15% of near occlusions, whereas most were MeVOs. Thus, no firm conclusions can be drawn about efficacy of IVT for patients with LVO and minor symptoms. Instead, our results align with the recent RCTs on MeVO, where endovascular thrombectomy did not improve outcome despite a recanalisation rate of 72%–75% in the intervention group.^21^^,^^22^ These findings suggest that recanalisation may not be as important a determinant of reduced disability in more distal occlusions as in LVO.

We found an interaction of age and treatment arm in the complete occlusion subgroup suggesting that tenecteplase might be harmful for patients above 80 years compared to standard care, which aligns with another secondary analysis from the TEMPO-2 cohort.^23^ Additionally, interactions based on time from onset and sex in the complete occlusion group were observed; however, there was no significant difference between treatment arms within the early or late window group, nor among male or female patients.

The TEMPO-2 trial was a multicentre, randomised, blinded trial with central imaging assessment. Nonetheless, it was not powered to compare tenecteplase and control in subgroups with different occlusion status. Moreover, as the secondary outcomes of the present study were not adjusted for multiplicity, there is a risk of a chance finding. Hence, the results should be considered exploratory and interpreted cautiously. Although the subgroups were well balanced concerning their baseline characteristics, the few between-group differences create a risk of confounding bias. Additionally, the low number of patients with LVO or vertebrobasilar occlusion limits conclusions concerning optimal treatment of these subgroups. Non-thrombolytic therapy in the control arm comprised different antithrombotic strategies, which is a potential source of heterogeneity but also reflects clinical practice. Finally, we do not have data on how often patients with complete occlusion were treated outside the trial with reperfusion therapy due to assumed superiority, which impairs generalisability of the results.

In conclusion, the baseline vessel occlusion status did not explain the lack of benefit of IVT with tenecteplase vs standard care comprising mostly antiplatelet therapy for patients with minor AIS. Thus, the current evidence does not support the use of IVT for patients with minor stroke, independent of presence of vessel occlusion.

## Supplementary Material

Supplementary_material_aakag111

## Data Availability

Data collected for the study, including de-identified individual participant data and a data dictionary defining each field in the set, can be made available to others on reasonable request and after signing appropriate data sharing agreements. Please send data access requests to scoutts@ucalgary.ca. Such requests must be approved by all the respective ethics boards and appropriate data custodians.
