# Supplementary material for: Tenecteplase versus standard care for minor ischaemic stroke according to the baseline occlusion status in TEMPO-2 trial
Source: Eur Stroke J. 2026 Sep 14;11(9):aakag111. doi: 10.1093/esj/aakag111 (PMC13573578; doi:10.1093/esj/aakag111)
Supplement: Supplementary file 1 [file supplementary_material_aakag111.zip › Supplementary_material_TEMPO-2_investigators.docx]

**Acknowledgements: Central and by Sites**

**CENTRAL**

Calgary Image Processing and Analysis Centre: Marina Salluzzi, Nicole Blenkin, Ashley Dueck

Contracts and Finances: Craig Doram, Qiao Zhang

Site Monitors: Carol Kenney, Charlotte Zerna, Karla Ryckborst, Shelly Bohn

Safety Review Committee: P Barber, T Field, D Dowlatshahi

Data Safety Monitoring Board: Robert Hard (Chair), David Kent, Renee Martin and William Whiteley.

Website and Video: Quinten Collier

Clinical Research Unit: Frances Taylor, B. Cord Lethebe

**By Enrolling Sites:**

**Foothills Medical Centre, University of Calgary, Calgary, AB**

Site PI: PA Barber

Primary Study Coordinators: C Kenney, A Jambula,, K Sage, K Ryckborst, L Toussaint,, S Save , J Lee, N Laham

Other Site Investigators: A A Sultan, A Moussaddy, A Deepak, A Sitaram, A Y X Yu, A M Demchuk, A Lockey, A Micielli, A Wadhwa, A Ganesh, B Arabambi, B K Menon, B Graham, C Tham, C Zerna, C Bogiatzi, D Doshi, D Chakraborty, D Kim, D Vasquez, D Singh, D Tse, E Harrison, E Smith, E Teleg, E Klourfeld, G Klein, I A Sebastian, J Evans, J Hegedus, J Kromm, K Lin, K Ignacio, K Ghavami, M Ismail, M Moores, M A Panzini, M Marko, M Boyko, M D Hill, M A Almekhlafi, N Newcommon, N Maraj, N Singh, O Imoukhuede, O Volny, P Stys, P Barber, P Couillard, P Ojha, P Eswaradass, R Joundi, R Appireddy, R Singh, R M Asuncion, R T Muir, S Dey, S Mansoor, S Wasyliw, S Nagendra, S Hu, S Althubait, S Chen, S Bal, S Van Gaal, S Peters, S Ray, S Chaturvedi, S Subramaniam, V Fu

**Vancouver General Hospital, Vancouver, BC**

Site PI: T S Field

Primary Study Coordinators: K Villaluna, G Maclean, P King-Azote, C Ma,

Other Site Investigators: A Plecash, C Murphy, D Tse, J Gorman, K Ghavami (Also listed under Calgary), L Wilson, L Zhou, O Benavente, P Teal, S Yip, S Mann, S Van Gaal

**Ottawa Hospital Research Institute, Ottawa, ON**

Site PI: D Dowlatshahi

Primary Study Coordinators: B Dewar, M Demetroff, R Shamloul, R Beardshaw, S Roberts, Z Daham

Other Site Investigators: D Blaquiere, G Stotts, M Shamy, O Bereznyakova, R Fahed, W Alesefir

**Enfant-Jésus Hospital, Centre Hospitalier Universitaire de Québec, Quebec City, QC**

Site PI: M C Camden

Primary Study Coordinator: Suzy Lavoie, A Hache, K Collard,

Other Site Investigators: A Mackey, S Gosselin-Lefebvre, S Verreault

**London Health Sciences Centre, University Hospital, London, ON**

Site PI: J Mandzia

Primary Study Coordinators: B Beauchamp, L Lambourn

Other Site Investigators: A Khaw, L Mai, L Sposato, M Bres Bullrich, R Azarpazhooh, S Fridman

**Sunnybrook Health Sciences Centre, University of Toronto, Toronto, ON**

Site PI: A Y X Yu

Primary Study Coordinators: A Kapoor, A Southwell, E Bardi, I Fatakdawala, M Kamra, K Lopes, N Popel, V Norouzi

Other Site Investigators: A Liu, AM Liddy, B Ghoari, C Hawkes, C A Enriquez, D J Gladstone, H A Manosalva Alzate, H Khosravani, J J Hopyan, K Sivakumar, M Son, M I Boulos, M A Hamind, R H Swartz, R Murphy, S Reiter, T Fitzpatrick, V Bhandari

**Victoria General Hospital, Victoria, BC**

Site PI: A Penn

Primary Study Coordinators: J Good, M Penn, M Naylor, S Frost

**Toronto Western Hospital, University Health Network, University of Toronto, Toronto, ON**

Site PI: L Casaubon

Primary Study Coordinators: A Cayley, F Akthar, J Williams, L Kalman, L Crellin, R Wiegner, S Singh, T Stewart, W To, S Singh

Other Site Investigators: A Pikula, C Jaigobin, F Carpani, F Silver, H Janssen, J Schaafsma, K Sivakumar (Also listed under Sunnybrook), M del Campo, M Alskaini, P Rajendram

**University of Alberta Hospital, Edmonton, AB**

Site PI: B Buck

Primary Study Coordinators: P Fairall, B Granfield, D Crawford, J Jabs, L White, L Sivakumar, L Piquette, T Nguyen

Other Site Investigators: A Nomani, A Wagner, A Alrohimi, A Butt, A D'Souza, B Gajurel, C Vekhande, H Kamble, H Kalashyan, K Butcher, M Lloret, M Benguzzi, N Arsalan, N Ishaque, R Ashayeriahmadabad, R Samiento, S Mishra, S Hosseini, S Kazi, S Das, T Sugumar

**St. Michael's Hospital, Toronto, ON**

Site PI: A Zafar and D Selchen (also listed in SM as PI)

Primary Study Coordinators: P Kostyrko

Other Site Investigators: A Muccilli, G Saposnik

**Medical University of Vienna, General Hospital, Vienna, Austria**

Site PI: S Greisenegger

Primary Study Coordinators: K Werner, S Wieszmuellner,

Other Site Investigators: A Langer, A Gisold, H Zach, M Marko, P Rommer, S Macher, S Blechinger, W Marik, W Series

**St. John's of God Hospital, Vienna, Austria**

Site PI: J Ferrari

Primary Study Coordinators: M Baumgartinger

Other Site Investigators: S Krebs

**Hamilton Health Sciences Centre, Hamilton, ON**

Site PI: L Catanese

Primary Study Coordinators: C Vandervelde, K Ratnayake, S McMillan

Other Site Investigators: A Katsanos, A Shoamanesh, D J Sahlas

**Royal Columbian Hospital, New Westminster, BC**

Site PI: G Medvedev

Primary Study Coordinators: V Naidoo, V Todorov

Other Site Investigators: H Toma, J Brar, J Lee, M Horton, S Chen

**Red Deer Regional Centre Hospital, Red Deer, AB No DOA for site**

Site PI: O Imoukhuede

Primary Study Coordinators: E Shand

Other Site Investigators:

**Kingston General Hospital, Kingston, ON**

Site PI: R Appireddy

Primary Study Coordinators: S Weatherby

Other Site Investigators: A Jin, B Durafourt, S Jalini

**Royal University Hospital, Saskatoon, SK**

Site PI: G Hunter

Primary Study Coordinators: A Gardner, C Tyson, E Junk, K Foster, K Bolt, N Sylvain, S Maley

Other Site Investigators: B Graham, L Urroz, L Peeling, M Kelly, R Whelan, S Wasyliw, R Cooley

**McGill University Health Centre, Montreal, QC**

Site PI: A Moussaddy and J Teitelbaum (also listed as PI in SM)

Primary Study Coordinators: A Boutayeb, A Moore, E Cole, L Waxman, N Ben-Amor, R Sanchez, S Khalil

Other Site Investigators: A Nehme, C Legault, D Tampieri, E Ehrensperger, L Vieira, M Cortes, M Angle, M Hannouche, M Badawy

**Helsinki University Hospital, Helsinki, Finland**

Site PI: D Strbian

Primary Study Coordinators: J Koski, S Eirola, T Ivanoff, A Erakanto, L Kupari,

Other Site Investigators: G Sibolt, J Panula, L Tomppo, M Tiainen, M Ahlstrom, N Martinez Majander, O Suomalainen, S Raty

**John Hunter Hospital, New South Wales, Australia**

Site PI: C Levi and N Spratt

Primary Study Coordinators: E Kerr, J Allen, L P Kaauwai, L Belevski, M Russell, S Ormond

Other Site Investigators: A Chew, A Loiselle, A Royan, B Hughes, C Garcia Esperon, E Pepper, F Miteff, J He, M Lycett, M Min, N Murray, N Pavey, R Starling de Barros, S Gangadharan, S Dunkerton, S Waller, T Canento Sanchez, T Wellings

Site PI: D Ghia

Primary Study Coordinators: G Edmonds, K A Whittaker, M Ewing, P Lee, R Singkang

**Royal Melbourne Hospital, Parkville, Australia**

Site PI: B Campbell

Primary Study Coordinators: A McDonald, A Dos Santos, C Shin, D Jackson, J Tsoleridis, L Fisicchia, N Parsons, N Shenoy, S Smith

Other Site Investigators: A Sharobeam, A Balabanski, A Park, C Williams, D Pavlin-Premri, E Rodrigues, F Alemseged, F Ng, H Zhao, J Beharry, J L Ng, J Williamson, J Z W Wong, K Li, M K Kwan, M Parsons, M Valente, N Yassi, R Cooley, V Yogendrakumar

**Box Hill Hospital, Box Hill, Australia**

Site PI: P Choi

Primary Study Coordinators: B McNamara, C Buchanan, C McCarthy, G Thomas, K Stephens, M F Chung, M Tang, N Pachani, N Stuart, R Lee, T Frost, T Busch, M Chung

Other Site Investigators: A Menon, B Borojevic, C M Linton, E P Callaly, G Garcia, H Dewey, J Liu, J Chen, J Wong, K Nowak, K To, N S Lizak, O Bhalala, P Park, P Tan, R Martins, R Cody, R Forbes, S K Chen, S Tu, S Ooi, Y L Dang, Z Ling

**Royal Adelaide Hospital, Adelaide, Australia**

Site PI: T Kleinig

Primary Study Coordinators: J Cranefield, R Drew

Other Site Investigators: A Tan, C Kurunawai, J Harvey, J J Mahadevan, L Cagi, L Palanikumar, N Chia, R Goh, S El-Masri

**Gold Coast University Hospital, Gold Coast, Australia**

Site PI: P Bailey, S Mishra

Primary Study Coordinators: B Urbi, C Rapier, H Berrill, H McEvoy, R Dunning, S Kuriakose, T Chad, V Sapaen

Other Site Investigators: A Sabet, D Shah, D Yeow, K Lilley, K Ward, M Mozhy Mahizhnan, M Tan

**Mater Misericordiae University Hospital, Dublin, Ireland**

Site PI: Peter Kelly

Primary Study Coordinators: C Lynch, S Coveney, K Tobin

Other Site Investigators: J McCabe, M Marnane, S Murphy

**Beaumont Hospital, Dublin, Ireland**

Site PI: D Williams

Primary Study Coordinators: M Large

Other Site Investigators: B Moynihan, K Boyle

**Hospital Universitari Vall d'Hebronq, Barcelona, Spain**

Site PI: C Molina

Primary Study Coordinators: E Sanjuan ,M Sanchis, O Pancorbo, V Sala, L Garcia,

Other Site Investigators: A Garcia-Tornel, , J Juega, J Pagola, K Santana, M Requena, M Muchada, M Olive, M Rubiera, M Deck, N Rodriguez, P J Lozano, S Boned

**Clinic University Hospital Valladolid, Valladolid, Spain**

Site PI: J F Arenillas

Primary Study Coordinators: B Gomez, F J Reyes Munoz

Other Site Investigators: A S Gomez, A C Sanz, E C Garcia, G Penacoba, M E Ramos, M de Lera Alfonso

**Hospital Universitari Doctor Josep Trueta, Girona, Spain**

Site PI: J Leal

Primary Study Coordinators: A Feliu, L Pardo, P Ramirez

Other Site Investigators: A Murillo, D Lopez Dominguez, J Rodriguez, M Terceno Izaga, M Reina, S B Viturro, U Bojaryn, V A Vera Monge, Y Silva Blas

**National Neuroscience Institute, Tan Tock Seng Hospital, Novena, Singapore**

Site PI: C Tham

Primary Study Coordinators:R Siew, S J Agustin

Other Site Investigators:, C Seet,T Tianming

**Christchurch Hospital, Christchurch, New Zealand**

Site PI: T Y H Wu

Primary Study Coordinators: A d'Emden

Other Site Investigators: None listed

**Queen Elizabeth University Hospital, Glasgow, Scotland**

Site PI: K Muir

Primary Study Coordinators: A Murray, A Welch, K Hatherley, N Day, W Smith , E MacRae, S Mitchell

Other Site Investigators: A Sitaram, A Mahmood, J Elliot, S Neilson, V Biswas ,C Brown

University College London Hospital, London, England

Site PI: R Simister

Primary Study Coordinators: A Lewis, A Ashton, A Black, A Robinson, A Williams, A Banaras, C Cahoy, G Raingold, M Marinescu, N Atang, N Bason, N Francia, R Muhammad, S Obarey, S Feerick, Y C Lee

Other Site Investigators: D Werring, R Perry

**John Radcliffe Hospital, Oxford, England**

Site PI: J Kennedy

Primary Study Coordinators: J Joseph, J Benjamin, L Quinn, M Jhoots, R Teal

Other Site Investigators: G Ford, G Harston, H Bains, I Gbinigie, P Mathieson, R Irons, U Schulz

**St. George's University Hospital (Foundation Trust), London, England**

Site PI: B Clarke

Primary Study Coordinators: C H Sim, E Hayter, K Kennedy, L Binnie, N Priestley, R Williams, R Ghatala, S Stratton

Other Site Investigators: None listed

**Countess of Chester Hospital, Chester, England**

Site PI: K Chatterjee

Primary Study Coordinators: A Davies, H Duffy, J Roberts, J Homer, K Roberts, K Dodd, K Cawley, M Martin, S Leason, S Cotgreave, T Taylor

Other Site Investigators: A Nallasivan, S Haider, T Chakraborty, T Webster

**Charring Cross Hospital, London, England**

Site PI: O Halse

Primary Study Coordinators: A Gil, B Martin, B Joseph, C Cabrera, D Jose, J Man, J Aquino, L Sebastian, M Osterdahl, M Kwan, M Matthew, N Ike, P Bello, P Wilding, R Fuentes, R Shah, S Mashate, T Patel, U Nwanguma, V Dave

Other Site Investigators: A El-Masry, A Ali Sheikh, A C Dawson, A Haber, A Lee, A O’Sullivan, B Drumm, C O’Hare, D Roberts, D Kalladka, E Taylor, E Rounis, F Vonberg, I H Jenkins, J Blagojevic, J George, J Kwan, M Saeed, M Evans, M Haji-Coll, M Tsuda, M Sayed, N Thanbirajah, N Winterkron, O Raha, O Vittay, R Karim, R C Smail, S Gauhar, S Kalam, S Elmamoun, S Malani, S Pralhad Kelavkar, S Jamil, S Auger, T Matar, V Biswas

**University Hospital of North Midlands, Stoke, England**

Site PI: C Roffe

Primary Study Coordinators: J Hiden, R Varquez

Other Site Investigators: P Ferdinand, R Sanyal

**Royal Victoria Hospital, Belfast, Ireland**

Site PI: I Wiggam

Primary Study Coordinators: B Smith, C Okechukwu, E Fox, E Collins, K Courtney, S Tauro

Other Site Investigators: C Patterson, D McShane, E Kerr, G Roberts, J McIImoyle, K McGuire, P Fearon, P Gordon

**Birmingham City Hospital, Birmingham, England**

Site PI: M Willmot

Primary Study Coordinators: K Isaacs, K Lucas, L Smith, L Dews, M Bates, S Lawrence, S Heeley, V Patel, Y M Chin

Other Site Investigators: D Sims, E Littleton, J Khaira, K Nadar

**King's College Hospital, London, England**

Site PI: S Ankolekar

Primary Study Coordinators: A Kieliszkowska, B Sari, C Domingos Belo, E Smith, E Y Manolo, J Aeron-Thomas, M Doheny, M Garcia Pardo, M Recaman, M C Tibajia, M Aissa, S Bayhonan, S Conway

Other Site Investigators: A Bhalla, A Engineer, D Nouvakis, E Theochari, F Boyle, J Teo, J King-Robson, K Y Law, L Sztriha, M Ismail, M Benger, M Farag, O Williams, R Alsukhni, S Heller, S Meenakshisundaram, T Yu, V Patel, Y Mah

**Cambridge University Hospital, Cambridge, England**

Site PI: G Zachariah

Primary Study Coordinators: A McGovern, A Iqbal, D Day, J Mitchell-Douglas, J Francis, P Punjabivaryani

Other Site Investigators: J Anonuevo Reyes, M Pauls, M Anonuevo Reyes

**Nottingham City Hospital, Nottingham, England**

Site PI: K Krishnan

Primary Study Coordinators: A Buch, A Hedstrom, C Hutchinson, C Kirkland, G Wilkes, J Newham, L Fleming

**Porto Alegre Hospital, Porto Algre, Brazil**

Site PI: S Martins

Primary Study Coordinators: N Fleck

Other Site Investigators: A Franca, A Pille, B Chwal, C Oldoni, G Mantovani, G Noll, L Zanella, M Soma, T Secchi, W Borelli

**Hospital de Clinicas de Ribeirão Preto, Ribeirão Preto, Brazil**

Site PI: O Marques Pontes Neto

Primary Study Coordinators: B P Rimoli, G H da Cunha Silva, L A Machado Galvao Mondin, R Barbosa Cerantola

Other Site Investigators: A K Imthon, A S Esaki, A L A de Albuquerque, A M Pazini, C E Massote Fontanini, C F Matinez Rubio, C Milani, D T dos Santos, F A Dias, F F A Alves, G G Riccioppa Rodrigues, K Santos Ferreira, M A Pena Pereira, M B Morillos, M Camilo, O C Vincenzi, R R ds Cruz

**Hospital de Clinicas de Botucatu, Botucatu, Brazil**

Site PI: R Bazan

Primary Study Coordinators: B Pegorer Santos, F Winckler, J T De Souza, L A M Bonome, N C Ferreira,

Other Site Investigators: D F Barbosa dos Santos, G P Modolo, J C dos Santos Moreira, R S Teodoro, V A Cury Silva

**Hospital Geral de Fortaleza, Fortaleza, Brazil**

Site PI: F O Lima and A B Cruz Guedes de Morais (two PIs listed – not sure of order or second name)

Primary Study Coordinators: J Vieira

Other Site Investigators: G Mendes, J P de Queiroz

**Hospital Sao Paulo UNIFESP, San Paulo, Brazil**

Site PI: G Sampaio Silva

Primary Study Coordinators: A Haro, L Donato, T Rodrigues

Other Site Investigators: A C De Almeida, A E Vayego Fornazari, A F de Araujo Netto, C M da Silva, E Dos Santos Sousa, E Navarro Borba Adissy, G Manginelli Lamas, I Viera Terehoff, Ingrid Demosthenes Wanzileu, L Pacheco Avezum, M E Nunes Silva, M Trombin Marques, S J Silva Rolindo, V C Colares Lessa

**Neurocirurgica de Joinville LTD, Joinville, Brazil**

Site PI: C H Cabral Moro

Primary Study Coordinators: A P de Oliveira Pire, F M Vicente, L Gaio, N Bar Silva, R Bitencourt Liberato, S Aparecide da Silva Ribeiro, T dos Santos

Other Site Investigators: A L Longo, B H de Oliveira Marques da Silva, C M Marin, C Figueiredo da Silva, D L de Albuquerque, E M Leal, F Romancini, F W Dias Silva, G Schmitt Trevisan, G da Silva Miranda, H P Macedo Nunes, K Machado Lima, L Koenig de Lima, L Danielli, L P Barros de Moraes, M Silvio Gomes, M A Baptista Guimbala, M C Albino Patino, M E A de Mendonca Fileti, N Cardoso Darabas, P Silva Correa de Magalhaes, R da Silva Almeida Santos
